# Epidemiology of Cefotaxime-Hydrolysing β-Lactamase-Producing *Escherichia coli* in Children with Diarrhoea Reported Globally between 2012 and 2022

**DOI:** 10.3390/microorganisms12010171

**Published:** 2024-01-15

**Authors:** Simbarashe Karambwe, Afsatou Ndama Traoré, Natasha Potgieter

**Affiliations:** Department of Biochemistry and Microbiology, University of Venda, Private Bag X5050, Thohoyandou 0950, South Africa; karambwesim@gmail.com (S.K.); afsatou.traore@univen.ac.za (A.N.T.)

**Keywords:** *Escherichia coli*, diarrhoea, children, CTX-M-producing *E. coli*

## Abstract

The global spread of cefotaxime-hydrolysing β-lactamase (CTX-M)-producing *Escherichia coli* (*E. coli*) and its associated impact on paediatric diarrhoeal treatment and management has become a public health concern. This review assessed surveillance studies on CTX-M-producing *E. coli* associated with diarrhoea in children published between 2012 and 2022 globally. A total of thirty-eight studies were included for data analysis, categorised into continental regions, and tabulated. The majority (68%) of studies were conducted in Asian countries while few studies were conducted in Europe (11%) and Africa (18%), respectively. On the African continent, the majority (11%) of studies were conducted in Northern Africa while no studies were reported in East Africa. On the American continent, 3% of the studies were reported from South America. The studies included were classified into diarrheagenic *E. coli* (74%; 28/38) and faecal carriage (26%; 10/38). Of all the *E. coli* pathotypes associated with CTX-M production, EPEC was frequently reported. The prevalence of CTX-M-producing *E. coli* including the CTX-M-15-producing variants ranged between 1% and 94%. About 37% of the studies generalised the report as *bla*_CTX-M-_positive *E. coli*. The use of sequencing in characterising the CTX-M-producing *E. coli* was reported in only 32% of all the studies. This review provides information on the epidemiology of CTX-M-15-producing *E. coli* in paediatric diarrhoea and the extent to which surveillance is being performed. This is relevant in informing clinical practice for the management of diarrhoea as well as the design of future surveillance studies.

## 1. Introduction

The Global Burden of Disease, Injuries, and Risk Factors Study (GBD) ranked diarrhoea as one of the prime causes of death and disability-adjusted life years (DALYs) for children younger than 5 years. In 2016 alone, close to half a million deaths in children under 5 years were due to diarrhoea [1]. Asia, Africa, and America are among the continents that have reported high rates of deaths of children under two years of life due to diarrhoea [2].

Pathogenic strains of *Escherichia coli* (*E. coli*) are one of the causes of diarrhoea in children in developing countries [3]. These *E. coli* strains with diarrhoea-causing properties are known as diarrheagenic *E. coli* (DEC). There are six DEC pathotypes namely, enteropathogenic *E. coli* (EPEC), enteroaggregative *E. coli* (EAEC), enterotoxigenic *E. coli* (ETEC), enterohemorrhagic *E. coli* (EHEC) also known as Shiga toxin-producing *E. coli* (STEC), enteroinvasive *E. coli* (EIEC) and diffusely adherent *E. coli* (DAEC) [3,4]. Of the six pathotypes, EAEC, EPEC, and ETEC are the most common causes of diarrhoea episodes in children under five years in developing countries [5]. Diarrheagenic *E. coli* has also been characterised into phylogroups such as A, B1, B2 and D. Phylogroups A and D are mostly associated with diarrhoea [6,7] and human faecal matter is a possible source of DEC in these two phylogroups [8]. However, the association of DEC and phylogroups varies geographically [8].

According to the World Health Organisation (WHO), paediatric diarrheal infection in low-income countries is not only a risk to public health, but it is becoming increasingly untreatable due to emerging antibiotic-resistant patterns against commonly prescribed antibiotics [9]. Antibiotic resistance among diarrheagenic *E. coli,* which has spread across developing countries, has been associated with the overuse of antibiotics [4]. Travelling has also been implicated as another key driving factor facilitating the global spread of antibiotic resistance [10,11].

Cefotaxime (CTX) is a broad-spectrum cephalosporin antibiotic normally used in treating infections caused by bacteria resistant to first-line antibiotics. Cefotaxime-hydrolysing β-lactamases (CTX-M) which together with some variants of Temoneira (TEM) and sulphydryl variable (SHV) enzymes, are considered the most clinically significant beta-lactamases with extended-spectrum activity (ESBLs) [12,13]. Unlike TEM and SHV genes, which also have variants that exhibit non-ESBL characteristics, all CTX-M types are exclusively ESBL genes [14]. The CTX-M gene variants are not closely related to the most isolated β-lactamases, TEM and SHV genes [13]. Of the ESBLs, the CTX-M variants are leading in terms of spread and their impact is either comparable or even greater to that of Temoneira (TEM) and sulphydryl variable (SHV) ESBLs [15]. There are several CTX-M variants grouped into sub-families, CTX-M group 1, 2, 8,9, 25 and 45 among others [15]. Group 1 CTX-M variants are the most widespread globally compared to other variants and in Africa and Asia, reports indicated that CTX-M group 1 are more common [10,15]. Of the CTX-M Group 1, CTX-M-15 is currently the dominant variant and a cause of concern in clinical practice [16]. The literature suggests that the CTX-M-15 gene variants are widely distributed in countries in Europe, North and South America as well as Asia [17]. It is important to note that the prevalence of CTX-M-producing *E. coli* varies between regions [10,18]. The prevalence rates of CTX-M-producing *E. coli* are at least 60% in Asia [10] while a lower rate of 34% has been reported in West Africa [18].

Specific DEC pathotypes such as EPEC, ETEC and EAEC have been implicated among extended-spectrum beta-lactamases (ESBL) CTX-M (cefotaxime resistant) producers [4,19]. *E. coli* strains producing ESBLs such as CTX-M are a threat to public health and can exhibit co-resistance to other classes of antibiotics such as aminoglycosides and fluoroquinolones [20,21,22]. Information regarding specific *E. coli* pathotypes associated with CTX-M genes is scarce [19]. While CTX-M are the predominant ESBL genes encountered, two other genes, namely TEM, and SHV, which encode enzymes that confer beta-lactam resistance are also encountered in the *Enterobacteriaceae* group such as *E. coli* [16,18].

Diarrhoea in children under 5 years is implicated among the risk factors for acquiring ESBL-producing *E. coli* [23]. CTX-M-producing *E. coli* associated with diarrhoea cases in young children has been mostly reported in Asian countries [10] while antimicrobial resistance (AMR) surveillance in other regions such as Africa is slow or rather underreported due to limited resources and infrastructure [18]. Despite studies that investigated CTX-M-producing *E. coli* in diarrhoea cases in Africa [12,23,24,25,26], there is a dearth of information on beta-lactamase (CTX-M) resistance in *E. coli* associated with diarrhoea in young children. In addition, detailed genomic studies using sequencing techniques to uncover the epidemiology of high-risk clones such as sequence type 131 (ST131), which are associated with the dissemination of CTX-M genes are limited in Africa [27]. Previous studies that have been conducted in children investigated CTX-M-producing *E. coli* recovered from urinary infections [28]. This narrative review aimed to give an update on the reported prevalence of CTX-M-producing *E. coli* recovered from children less than 5 years of age with diarrhoea, especially tracing the epidemiology of the CTX-M-15 gene variant in the literature published between 2012 and 2022. This is relevant in understanding the local and regional epidemiology of CTX-M-producing *E. coli*, which is essential in guiding interventions and antimicrobial stewardship.

## 2. Methodology

### 2.1. Search Strategy and Selection Criteria

**Figure 1:**
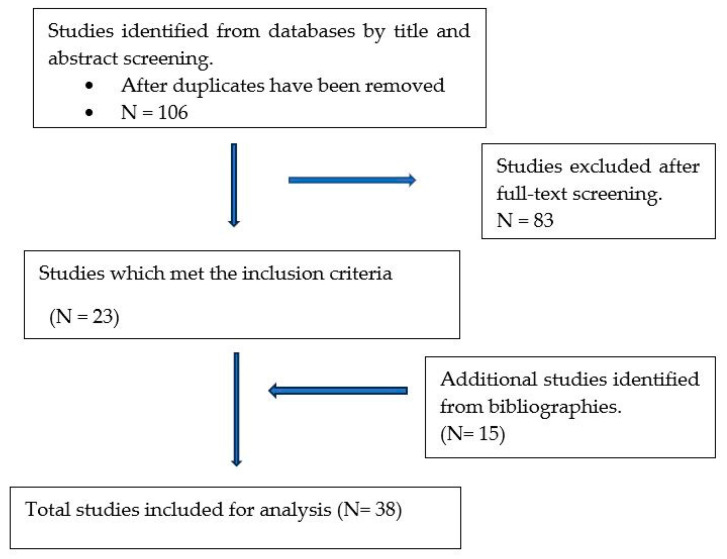
Flow diagram showing the filtering process followed on selection of studies.

A literature search was conducted for studies published between 1 January 2012 and 31 September 2022 using PubMed, Web of Science, Google Scholar and Science Direct databases. The following keywords were used: “*Escherichia coli*” OR “*E. coli*” and “CTX-M beta-lactamase” OR “CTX-M β-lactamase” OR “*bla*CTX-M” OR “CTX-M” AND “diarrhoea” OR “diarrhea”. The literature search was restricted to the following: last decade 2012–2022, studies on humans, age group 5 years and under and studies published in English language. In addition, supplementary literature search was carried out using the bibliographies of studies relevant to the objective of this study (Figure 1). The studies were thoroughly screened based on the title and the abstracts reporting on CTX-M-producing *E. coli* (Figure 1). For studies to be included in this review, both phenotypic and genotypic resistance must have been reported.

### 2.2. Data Categorisation

Data on the author name, publication year, study period, country, continent, identified gap, age group, study design (prospective or retrospective), sample size, study setting (hospital, community), method of detection (^1^*E.coli* and ^2^ESBL genes), causative organism (*E. coli* or *E. coli* pathotypes or *E. coli* phylogroups), the percentage of CTX-M genes reported, other ESBLs genes such as TEM, SHV and OXA among others, and most common ESBLs were extracted and entered into an excel spreadsheet (Supplementary File S1).

### 2.3. Data Analysis

Python programming language (Version 3.8.8) was used for data analysis. Python Libraries used included Pandas, a package used for storing and manipulating data and data visualisation libraries such as Matplotlib and Seaborn [29]. Analysis was limited to descriptive statistics.

## 3. Results

### 3.1. Causative Organism and Study Setting

A total of 38 studies were included in the analysis. The studies were grouped into two, diarrheagenic *E. coli* (28/38) (Table 1) and faecal carriage (10/38) (Table 2). Generally, *E. coli* isolates recovered from stool samples were characterised into pathotypes or phylogroups by PCR and/or a combination of PCR and serotyping. ESBL genes were also characterised using PCR and sequencing (Table 1 and Table 2). Of the studies that reported on the specific *E. coli* pathotypes, the distribution of pathotypes was as follows: EPEC (82%; 23/28), EAEC (53.6%; 15/28), ETEC (35.7%; 10/28), EIEC (21%; 8/28), EHEC (10.7%; 3/28), STEC (10.7%; 3/28) and none of the studies reported on DAEC pathotype (Table 1). Of the 23 studies that reported on EPEC pathotype, 3/23 studies provided details of typical (tEPEC) and atypical EPEC (aEPEC) [24,30,31].

Most studies on faecal carriage (6/10), generalised the causative organism as *E. coli* while 4/10 studies characterised *E. coli* based on the four phylogroups A, B1, B2 and D (Table 2). Phylogroups A, B1 and D were the most prominent [7,28,32,33] (Table 2).

Most of the studies were conducted at hospitals (25/38), followed by primary healthcare centres (6/38) and community settings (2/38). Only a few studies have specified the geographical settings as either urban (13%; 5/38 studies) or rural (5%; 2/38 studies) (Table 1 and Table 2).

**Table 1:** Summary of studies on *bla*CTX-M-15-producing diarrheagenic *E. coli* recovered from children with diarrhoea across different continents.

Country and Continent	Setting	Design	Age Group	Sample Size	Detection Methods (^1^*E. coli* and/or Pathotype, ^2^ESBL Genes)	Causative Organism	% *bla*_CTX-M_ Reported	CTX-M Genes Detected	Other ESBLs Genes Detected	Study Period	Reference
Brazil, South America	ND	Case–control	0–5	162	^1^PCR, ^2^PCR	EPEC, EAEC	15	CTX-M	TEM		[34]
Egypt, North Africa	Hospital	Prospective	0–5	113	^1^PCR, ^2^Sequencing	EAEC	4.0	CTX-M	TEM	2016	[35]
Egypt, North Africa	Hospital	Prospective	0–5	320	^1^m-PCR, ^1^phylogrouping, ^2^PCR	EAEC, tEPEC, aEPEC	37.5	CTX-M-15	TEM	2018–2019	[24]
Burkina Faso, West Africa	Health centre	Retrospective	0–5	ND	^1^m-PCR, ^2^m-PCR	EPEC, EAEC	7.1	CTX-M	OXA	2018–2019	[23]
Libya, North Africa	Hospital	Prospective	0–5	290	^1^m-PCR, ^2^m-PCR	EAEC, EIEC, EHEC	60	CTX-M-15	CTX-8, CTX-M9	2012	[36]
England, Europe	Primary healthcare	Retrospective	0–16	660	^1^PCR, ^1,2^Sequencing	EAEC, ETEC, EPEC, EIEC	ND	CTX-M-15	TEM1, CTXM1, CTX-M14, CTX-M27, SHV12	2015–2017	[37]
India, Asia	Hospital	Prospective	0–5	120	^1^m-PCR, ^2^Rt-PCR, ^2^Sequencing	EPEC, EAEC, ETEC, EHEC	40	CTX-M	TEM, SHV, OXA, NDM-1, IMP, VIM, ACT, DHA and CMY	ND	[38]
Korea, Asia	Hospital	Prospective longitudinal	Children and infants	ND	^1^m-PCR, ^2^m-PCR	EPEC, ETEC, EHEC	16	CTX-M-15	CTX-M14, CTX-M27, CTX-M55, CTX-M3, TEM1, PABLs, CMY2, DHA1	2007–2016	[39]
Iran, Asia	Hospital	Descriptive cross-sectional study	0–5	321	^1^m-PCR, ^1^serotyping, ^2^PCR	EPEC	83.3	CTX-M	TEM	2016–2017	[40]
Iran, Asia	Hospital	Prospective	0–92	340	^1^PCR, ^2^PCR	STEC	69	CTX-M-9	TEM	2014	[41]
Qatar, Asia	Hospital	Prospective	0–10	175	^1^PCR, ^2^PCR	EPEC, EAEC	88.2	CTX-M-15	CTX-M-3	2017–2018	[42]
Iran, Asia	ND	Prospective	0–10	1355	^1^PCR, ^2^PCR	EPEC	10.9	CTX-M	TEM, SHV, OXA	ND	[20]
China, Asia	Hospital	Prospective	0–5	684	^1^PCR, ^1^Serotyping, ^2^PCR, ^2^Sequencing	EPEC, EAEC, ETEC, EIEC, STEC	20	CTX-M-15	NDM1, KPC2, TEM1, CTX-M-55, CTX-M14, CTXM-65, CTX-M-137	2015–2016	[3]
Iran, Asia	Hospital	Prospective	0–15	395	^1^PCR, ^1^phylogrouping, ^2^PCR	ETEC, EPEC	ND	CTX-M	TEM	2014–2015	[43]
India, Asia	Paediatric institute	Prospective and retrospective	0–10	900	^1^PCR, ^1^Serotyping, ^2^PCR	tEPEC, aEPEC	11.5	CTX-M-15	(NDM-1), (VIM)	2012–2013	[30]
Indonesia, Asia	Hospital	Prospective	0–3	133	^1^PCR, 2PCR, ^2^Sequencing	EAEC, EPEC	84	CTX-M-15	TEM-1, SHV	2012	[44]
India, Asia	Hospital	Cross-sectional study	0–5	120	^1^PCR,^2^PCR	tEPEC, aEPEC, ETEC, EIEC	ND	CTX-M	SHV, TEM	2015–2016	[31]
Pakistan, Asia	ND	Cross-sectional	0–5	100	^1^PCR, ^1^Sequencing, ^2^PCR	EPEC	93	CTX-M	TEM	2016–2017	[45]
Japan, Asia	Clinics	Retrospective	ND	167	^1^PCR, ^1^Phylogrouping, ^2^PCR, ^2^Sequencing	EAEC	79	CTX-M-15	CTX-M14, CTX-M55	1992–2010	[46]
India, Asia	Hospital	Prospective longitudinal	0–14	8891	^1^m-PCR, ^2^PCR	ETEC, EAEC, EPEC	30.2	CTX-M3	TEM, SHV, OXA1	2012–2019	[47]
Iran, Asia	Hospital	Prospective	0–10	303	^1^m-PCR, ^2^PCR	EAEC, EPEC, ETEC, EIEC, STEC	25	CTX-M-15	TEM	2018	[48]
China, Asia	Hospital	Prospective	0–5	1643	^1^PCR, ^1^Serotyping, ^2^PCR, ^2^Sequencing	EPEC	60.3	CTX-M-1	CTX-M9, TEM, SHV	2009	[49]
Iran, Asia	Hospital	Descriptive cross-sectional study	0–81	581	^1^PCR, ^2^PCR	EIEC	77.8	CTX-M-15	CTX-M1, TEM1	2016–2017	[50]
China, Asia	ND	Prospective	ND	912	^1^PCR, ^2^PCR, ^2^Sequencing	ETEC, EPEC, EIEC, EAEC	ND	CTX-M-14	CTX-M79, CTX-M28, TEM	2013–2014	[51]
Iran, Asia	Hospital	Prospective longitudinal	0–10	342	^1^PCR, ^1^Serotyping, ^2^PCR	EPEC	19	CTX-M-15	TEM, SHV	2011–2013	[4]
Iraq., Asia	ND	Prospective	0–2	656	^1^Serotyping, ^2^PCR	EPEC	77.3	CTX-M	TEM, SHV, OXA, AmpC	2009	[52]
Iran, Asia	Referral centre	Prospective	0–14	230	^1^PCR, ^1^Serotyping, ^2^PCR	EAEC, EPEC, EIEC, ETEC	94.4	CTX-M-15	TEM, AmpC	2015–2016	[53]
Iran, Asia	Hospital	Prospective	0–10	251	^1^PCR, ^1^Serotyping, ^2^PCR	EPEC	70.6	CTX-M-15	TEM	2015–2016	[54]

ND = no data; DEC= diarrheagenic *E. coli*; EPEC = enteropathogenic *E. coli*; EAEC = enteroaggregative *E. coli*; PCR = polymerase chain reaction; Rt-PCR = real-time PCR; m-PCR = multiplex PCR; tEPEC = typical; aEPEC = atypical; detection methods; Superscript 1 = method for *E. coli* detection, Superscript 2 = method for ESBL detection.

**Table 2:** Summary of studies on faecal carriage of *bla*CTX-M-15-producing *E. coli* recovered from children with diarrhoea across different continents.

Country and Continent	Setting	Design	Age Group	Sample Size	Detection Methods (^1^*E. coli* and/or Pathotype, ^2^ESBL Genes)	Causative Organism	% *bla*_CTX-M_ Reported	CTX-M Genes Detected	Other ESBLs Genes Detected	Study Period	Reference
South Africa, Sub-Saharan Africa	Community	Prospective longitudinal	0–1	65	^1^Cuture, ^2^PCR, ^2^Sequencing	*E. coli	4.9	CTX-M-14	TEM-1, CTX-M-9	ND	[12]
Nigeria, West Africa	Hospital	Prospective	0–5	296	^1^Culture, ^2^PCR, ^2^Sequencing	*E. coli	73.3	CTX-M	TEM, SHV	ND	[55]
Libya, North Africa	Clinics	Prospective longitudinal	3–12	243	^1^Culture, ^1^Phylogrouping, ^2^PCR, ^2^Sequencing	DEC: phylogroup B1, D, A and B2	13.4	CTX-M-15	CTX-M1, CTX-M3, TEM, SHV, OXA	2001 and 2007	[28]
France, Europe	Hospital	Prospective	0–16	1118	^1^Culture, ^2^PCR, ^2^Sequencing	*E. coli	4.3	CTX-M-15	TEM-24, TEM-19, SHV-5	2010–2011	[56]
Italy, Europe	Community	Prospective	0–6	482	^1^Culture, ^1^Phylogrouping, ^2^PCR, ^2^Sequencing	DEC: Phylogroup A, B1 and D	43	CTX-M	CTX-M1, CTX-M9, CTX-M8, CTX-M2	2011	[33]
Poland, Europe	Hospital	Prospective	0–5	ND	^1^Phylogrouping, ^2^PCR	DEC: Phylogroup A, B1, B2 and D	76.6	CTX-M	TEM, SHV	2008–2009	[7]
Iran, Asia	Hospital	Prospective	0–80	216	^1^m-PCR, ^1^phylogrouping, ^2^PCR	DEC: phylogroup A, D, B1 and B2	25.9	CTX-M-15	OXA1	2013	[32]
Iraq, Asia	Hospital	Prospective cross-sectional	0–8	116	^1^PCR, ^2^PCR	DEC	71.4	CTX-M	TEM-1	2019	[2]
Jordan, Asia	Hospital	Prospective	0–1	288	^1^Culture and Biochemical test, ^2^PCR, ^2^Phylogrouping	*E. coli	73.2	CTX-M-15	ND	2012	[57]
Malaysia, Asia	Hospital	Prospective	0–5	110	^1^Culture, ^2^PCR	*E. coli	9.1	CTX-M-15	TEM-1, CMY-2	2009–2010	[58]

ND = no data; DEC= diarrheagenic *E. coli*; PCR = polymerase chain reaction; m-PCR = multiplex PCR; *E. coli = *E. coli* not categorised as DEC; Superscript 1 = method for *E. coli* detection, Superscript 2 = method for ESBL detection.

### 3.2. Distribution of Studies on CTX-M-Producing E. coli by Region

**Figure 2:**
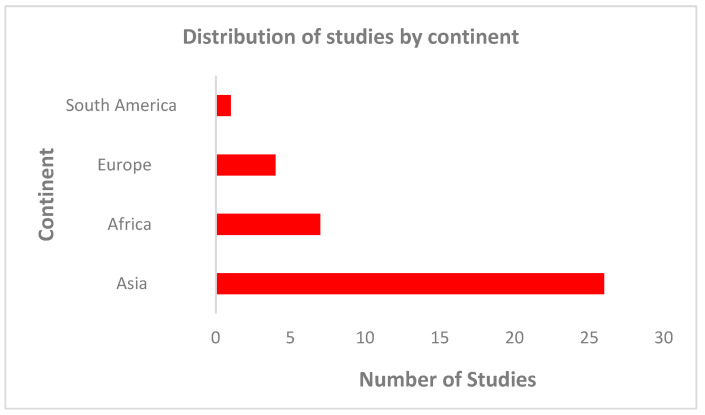
Distribution of studies conducted on CTX-M-producing E. coli in paediatric diarrhoea cases across continents.

Most of the studies on CTX-M-producing *E. coli* were conducted in countries in Asia (68%; 26/38) compared to studies found in European countries (11%; 4/38) and in countries on the African continent (18%; 7/38). Only one (3%) study was conducted in countries in South America (Figure 2). On the African continent, 11% (4/38) of the studies were conducted in North Africa, 5% of the studies were conducted in West Africa (2/38) and 3% (1/38) of the studies were conducted in Sub-Saharan Africa. In Asia, eight studies were reported from Iran, four studies were reported in India and three studies were reported in China (Table 1 and Table 2). Overall, faecal carriage studies were mostly reported in Europe and Africa, while most of the studies in Asia were mainly based on diarrheagenic *E. coli*.

### 3.3. Age Distribution

Only 36% (14/38) of the studies reported on the 0–5 years age group, 11% (4/38) of the studies assessed children under the age of 3 years; 40% (15/38) of the studies reported on the age groups between 0 and 16 years and 8% (3/38) of the studies investigated a mixed population between birth and 92 years of age. All the studies reporting a wide age range (0–100 years) were conducted in Iran and Western Asia (Table 1 and Table 2).

### 3.4. Distribution of Studies by E. coli Pathotype

Overall, about 21% (8/38) of studies reported specifically on EPEC. The prevalence of CTX-M producers among the EPEC-positive isolates ranged between 10 and 78%. Only two studies specified the existence of *bla*_CTX-M-15_-positive EPEC isolates (Table 3). Enteroaggregative *E. coli* (EAEC) was investigated in two studies in Asia (Japan) and North Africa (Egypt), respectively. The prevalence of CTX-M producers among the EAEC-positive isolates ranged between 19 and 50%. In both studies, only one CTX-M-15-producing EAEC isolate was observed among all the CTX-M producers [35,46]. On the other hand, STEC was only reported in one study conducted in Asia (Table 1).

### 3.5. Prevalence of CTX-M and Other ESBLs

In addition to the CTX-M gene variants, TEM was reported in 79% (30/38) of studies followed by SHV, which was reported in 34% (13/38) of the studies. Another ESBL, which was reported in 18% (7/38) of the studies was OXA, while CMY was reported in 8% (3/38) of the studies. Consequently, while 50% (19/38) of the studies reported on the CTX-M-15 variant, 37% (14/38) of the studies generalised the report as CTX-M. The other variants that were reported as part of the investigation included CTX-M-14 (5%; 2/38), CTX-M-9 (2%; 1/38), CTX-M-1 (2%; 1/38) and CTX-M-3 (2%; 1/38) (Table 1 and Table 2). Sequencing of the CTX-M gene was reported in 34% (13/38) of the studies (Table 1 and Table 2).

The prevalence of the CTX-M gene including the CTX-M-15 variant ranged between 1% and 94%, and the mean and standard deviation were 48% and 29%, respectively. The lowest prevalence rate was reported in Europe (1% and 4%). In Asia, the lowest rate (9%) was reported in Malaysia while the highest rate (94%) was reported in Iran. The mean rate of CTX-M-producing *E. coli* in Asia was 56%. Of the three common countries reporting on CTX-M-producing *E. coli* in Asia, the highest rate was reported in Iran (94%) followed by China (60%) and India (40%).

In the African continent, the prevalence of CTX-M-producing *E. coli* ranged between 5% and 73%, the lowest rate was reported in Sub-Saharan Africa (South Africa), while the highest rate was reported in West Africa. Most studies (4/7) in Africa were reported in North African countries, Egypt, and Libya. Only three studies reported CTX-M-15-producing *E. coli* associated with diarrhoea in children in Africa. It is evident that recent information on CTX-M-15-producing *E. coli* is scarce in Africa since only one study was conducted within the last 5 years between 2018 and 2019 [25]. Only one study confirmed the production of ESBL in isolates using the double disc synergy test [25] and only one study used sequencing [29]. There is a huge gap regarding standard approaches to surveillance due to resource constraints in Africa. Nevertheless, all three studies reported a low number of (8–15) CTX-M-15-producing *E. coli* isolates. Commensal isolates have been implicated as CTX-M-15 producers in one study [36] and thus *E. coli* is a prominent reservoir for ESBL genes.

In Europe, the literature on CTX-M-15-producing *E. coli* associated with diarrhoea in children is limited. Only two studies included in this review implemented sequencing to detail the epidemiology of CTX-M-15-producing *E. coli.* The prevalence of CTX-M-producing isolates ranged between 60% and 80%. In the studies included, the most common phylogroups were A, D and B1. The current review observed that CTX-M-producing *E. coli* was prominent among phylogroups A and D [7,28,32]. In addition, the CTX-M-15 variant was mostly associated with phylogroup D [28,32].

## 4. Discussion

This review describes the epidemiology of CTX-M-producing *E. coli* associated with diarrhoea in children based on studies published between 2012 and 2022. The prevalence of CTX-M gene varied between countries across the continents. The CTX-M gene was more common in Asian countries such as China, Iran and India and the highest prevalence (94%) of CTX-M was reported in Iran among MDR *E. coli* [53].

Most of the studies included in this review were conducted in clinical settings such as hospitals and clinics (Table 1 and Table 2). The impact of *E. coli* pathotypes such as EPEC and EAEC in causing hospitalisation of children suffering from diarrhoea has been reported [42]. In developing countries, EPEC is the leading cause of infantile diarrhoea [4]. The latter report explains the current observations in this review that EPEC was the most *E. coli* pathotype investigated for CTX-M resistance genes and to a lesser extent, CTX-M genes were also reported in EAEC and STEC. Thus, the tendency of EPEC and EAEC to carry CTX-M resistance genes is a cause of concern towards the management of diarrhoea in children because CTX-M-producing *E. coli* has been reported to be associated with increased resistance to first-line antibiotics, quinolone antibiotics as well as beta-lactam antimicrobials with an oxyimino side chain such as cephalosporins (cefotaxime, ceftriaxone and ceftazidime) and the oxyimino-monobactam (aztreonam) [33,59].

Understanding the epidemiology of CTX-M-producing *E. coli* is important in clinical practice. This review has shown that very few studies are being conducted in Africa on the surveillance of CTX-M-producing *E. coli* associated with diarrhoea in children. While Africa and Asia are flagged as regions with high morbidity and mortality rates in young children due to diarrhoea, it is important to uncover the epidemiology of antibiotic-resistant bacteria such as ESBL-producing *E. coli* that are more likely to complicate the treatment and management of diarrhoea in children. More studies on phenotypic resistance are conducted in developing countries whereas molecular surveillance of ESBL-producing *E. coli* is lacking [24]. The current review established that Sub-Saharan Africa, which is a hot spot of paediatric diarrhoea, is lagging regarding surveillance of CTX-M-producing *E. coli* unlike in Asia where such studies are being conducted across different regions. The literature suggests that Asian countries where at least 70% of the world population inhabits are epicentres for antimicrobial resistance [60]. A previous review in 2015 also reported that CTX-M-producing *E. coli* is the dominant multi-drug resistant (MDR) *E. coli* in Asian countries [10].

The most common CTX-M gene variant reported in North Africa was CTX-M-15 [28], which agrees with the findings of this study, especially in countries such as Egypt and Libya. No studies from East Africa were identified in this review. On the other hand, only one study from Southern Africa was identified. While East Africa and Southern Africa are key regions of Sub-Saharan Africa, which is known to experience the majority of childhood deaths due to diarrhoea [61], the current findings warrant more studies be conducted to understand the epidemiology of CTX-M-producing *E. coli* in paediatric diarrhoea cases in the region. Given that a previous review on the causes of gastroenteritis among children under 5 years in Sub-Saharan Africa reported that *E. coli* prevalence was high in the East Africa region [62], yet no study on CTX-M-producing *E. coli* was identified in this review, it is imperative to understand the antimicrobial resistance profile of such *E. coli* strains circulating in the East Africa region.

The current review did not find many studies in Europe on CTX-M-15-producing *E. coli* associated with diarrhoea in children. More studies were expected to be found as suggested by the literature that CTX-M beta-lactamases are more common in Europe [63]. However, the current insights may be explained by the fact that diarrhoeal diseases in children are less common in developed countries such as in Europe. The latter explains the observation in this review that most studies from Europe did not assess diarrheagenic *E. coli* but focused on the faecal carriage of *E. coli* instead. On the other hand, the literature highlights that in some countries, faecal specimens are not routinely tested for diarrheagenic *E. coli* [37]. The current observations that Europe together with America experience low rates of faecal colonisation by ESBL producers while Asia and Africa record high rates corroborates with results in a published review [64].

More often, CTX-M-15 has been associated with the co-production of other ESBLs such as TEM-1 and OXA-1 [64]. The findings in this review agree because TEM was reported more often (30/38) together with CTX-M-15, suggesting that TEM is one of the common ESBLs among faecal isolates. Molecular evidence on the mechanism of antibiotic resistance from a study in South Africa suggested that *TEM-1* is the main mechanism of beta-lactamase resistance in diarrheagenic *E. coli* [12]. On the other side, only 7/38 studies reported on the OXA-1 resistance genes (Table 1 and Table 2). Although OXA-1 genes are expected to be associated with CTX-M-producing *E. coli* [24], this study suggests that there is limited data available on the faecal carriage of OXA-1 genes.

## 5. Limitation

The present review focused only on studies that solely examined CTX-M-producing *E. coli* in paediatric diarrhoea. The inclusion criteria based on the availability of information on both phenotypic and genotypic resistance might have limited the number of studies included in the final analysis. The possible existence of other studies that reported on CTX-M-15 but co-investigated other pathogens together with *E. coli* is seen as a potential limitation in this study. The intention to describe the epidemiology of CTX-M-producing *E. coli* in the study period (2012–2022) might have reduced the number of studies. Some studies did not specify the exact variant but generalised the results as CTX-M. Given the continuous emergence of the CTX-M gene variants, efforts to conduct detailed molecular studies to characterise the CTX-M variants would provide information needed in clinical practice. Some of the studies included in this review did not specify the CTX-M-producing DEC strains, however, the report was generalised as DEC. The availability of such information is relevant in clinical practice as well as guiding the design of future studies.

## 6. Closing Remarks

This review showed that CTX-M-15-producing diarrheagenic *E. coli* has disseminated globally. However, there is a varying degree in the surveillance of CTX-M-15 in paediatric diarrhoea across continents and countries. The review showed that CTX-M-15-producing *E. coli* is common in Asian countries as well as in Northern and Western Africa regions. Integrated surveillance approaches are prominent in Asia, while there is a lack of recent studies in Africa, Europe and America. The dearth of detailed molecular studies in Africa, which is a hotspot for diarrhoea in children, warrants future research to help understand the role of CTX-M-15 in paediatric diarrhoea.

## Figures and Tables

**Table 3:** Summary of studies on CTX-M-producing Enteropathogenic *E. coli* (EPEC) recovered from paediatric diarrhoea cases.

No. of EPEC Isolates	Prevalence of CTX-M Producers (%)	Prevalence of *bla*_CTXM-15_	Reference
87	13 (15)	ND	[45]
59	7 (12)	7	[30]
58	31 (56)	ND	[49]
192	21 (11)	ND	[20]
22	17 (77)	ND	[52]
14	10 (71)	ND	[40]
42	8 (19)	8	[4]
17	12 (71)	ND	[54]

ND = No data on *bla*CTX-M-15.

## Data Availability

The data presented in this study are available on request from the corresponding author on reasonable request.

## Supplementary Materials

The following supporting information can be downloaded at: https://www.mdpi.com/article/10.3390/microorganisms12010171/s1, File S1: Database of studies on CTX-M-Producing *E. coli* between 2012 and 2022.
